# Treatment Patterns and Attrition in Metastatic Castration-Resistant Prostate Cancer

**DOI:** 10.1001/jamanetworkopen.2026.20750

**Published:** 2026-06-29

**Authors:** Gabriel Hooper, Yeonjung Jo, Varun Nandakumar, Georges Gebrael, Zeynep Irem Ozay, Micah Ostrowski, Tanner Hardy, Edwin Lin, Patrick Campbell, Ethan G. Murdock, Laxmi Upadhyay, Krishnam Goel, Vinay Mathew Thomas, Haoran Li, Alexandra O. Sokolova, Umang Swami, Neeraj Agarwal

**Affiliations:** 1 Division of Medical Oncology, Department of Internal Medicine, Huntsman Cancer Institute, University of Utah, Salt Lake City; 2 Division of Medical Oncology, Department of Internal Medicine, University of Kansas Cancer Center, Westwood; 3 Division of Hematology Oncology, Department of Internal Medicine, Oregon Health and Science University, Portland

## Abstract

**Importance:**

Rapid expansion of the therapeutic landscape for metastatic castration-resistant prostate cancer (mCRPC) has increased the complexity of treatment selection and sequencing. However, large, contemporary studies describing treatment patterns and attrition rates in clinical treatment of mCRPC are lacking.

**Objective:**

To investigate treatment patterns and attrition rates in patients with mCRPC from 2021 to 2025.

**Design, Setting, and Participants:**

This retrospective multicenter cohort study used the US-based, electronic health record–derived, deidentified Flatiron Health Research Database to identify patients with mCRPC who initiated first-line therapy between January 1, 2021, and June 30, 2025, at approximately 280 centers, primarily community oncology practices.

**Exposures:**

Year of first-line treatment initiation and types of treatment for mCRPC.

**Main Outcomes and Measures:**

First- through fifth-line treatment and attrition rates were summarized using frequencies and percentages into respective categories: androgen receptor pathway inhibitors (ARPIs), taxanes, poly (adenosine diphosphate–ribose) polymerase inhibitors (PARPIs), platinum-based therapies, lutetium Lu 177–prostate-specific membrane antigen 617 (Lu-177)–based therapies, radium Ra 223, sipuleucel-T, immunotherapy, and other.

**Results:**

Of 27 979 patients in the dataset, 5096 with mCRPC initiating first-line therapy were included in the analysis. The median age was 75 (IQR, 68-82) years. Overall, 2731 patients (53.6%) received second-line treatment and 1334 (26.2%) received third-line treatment. ARPI was the most common first-line (4041 of 5096 [79.3%]) and second-line (1123 of 2731 [41.1%]) treatment, with decreasing use in subsequent lines. Taxane use increased from 637 of 5096 patients (12.5%) in first-line treatment to 953 of 2731 (34.9%) in second-line treatment and became the most common third-line treatment (485 of 1334 [36.4%]). Overall, 2192 patients (43.0%) received taxane-based therapies. Use of Lu-177 increased progressively from 113 of 5096 patients (2.2%) in first-line treatment to 71 of 226 patients (31.4%) by fifth-line initiation. PARPI use was limited and peaked at 99 of 1334 patients (7.4%) in third-line treatment.

**Conclusions and Relevance:**

In this cohort study of patients with mCRPC, only slightly more than half of patients received second-line treatment, and attrition rose significantly across subsequent treatment lines. ARPIs and taxanes remained the most common first-line treatments, with increased use of Lu-177 and PARPIs in later treatment lines. These findings highlight the evolving complexity of mCRPC management and significant attrition with later lines, underscoring the need for optimized sequencing strategies, more efficacious frontline therapies, and interventions to improve access.

## Key Points

**Question:**

What are the current treatment patterns and attrition rates in metastatic castration-resistant prostate cancer (mCRPC)?

**Findings:**

In this cohort study of 5096 patients with mCRPC initiating first-line treatment between January 2021 and June 2025, 54% received second-line treatment and 26% received third-line treatment. Androgen receptor pathway inhibitors were the most common treatments in first and second lines, while taxanes became the most used option in later lines of treatment.

**Meaning:**

These findings suggest that there is a need for optimized sequencing strategies, more efficacious frontline therapies, and interventions to improve access to treatment for mCRPC.

## Teaser

This cohort study characterizes treatment patterns and attrition rates in a contemporary dataset of patients with metastatic castration-resistant prostate cancer to understand how these therapies are used in clinical practice.

## Introduction

Despite major advances in metastatic hormone-sensitive prostate cancer (mHSPC), most patients ultimately experience progression to metastatic castration-resistant prostate cancer (mCRPC), the lethal form of the disease.^1^ During the past 2 decades, the treatment landscape of mCRPC has expanded substantially. Treatment options include taxanes (docetaxel^2^ and cabazitaxel^3^), which were among the earliest treatments approved for mCRPC. From 2010 onward, therapies with additional mechanisms of action were added to the treatment armamentarium, including sipuleucel-T,^4^ androgen receptor pathway inhibitors (ARPIs) such as abiraterone acetate^5^ and enzalutamide,^6^ radium Ra 223,^7^ and pembrolizumab (for selected patients based on tumor genomic profile).^8^ Since 2020, advances in genomics and radioligand therapy have reshaped the therapeutic landscape with the introduction of poly (adenosine diphosphate–ribose) polymerase inhibitors (PARPIs) such as olaparib and rucaparib for patients with homologous recombination repair (HRR) mutations and BRCA1/2 alterations, respectively,^9,10^ and lutetium Lu 177–prostate-specific membrane antigen 617 (Lu-177) in patients with disease progression during prior ARPI and docetaxel treatment.^11^ More recently, combining PARPIs with ARPIs (olaparib plus abiraterone, niraparib plus abiraterone, and talazoparib plus enzalutamide) have been approved for selected patient populations,^12,13,14^ along with Lu-177 for taxane-naive patients who experienced progression while receiving prior ARPI treatment.^15^

These recent therapeutic advancements offer improved survival for patients with mCRPC but also introduce additional complexity to treatment decisions and sequencing. Clinical treatment patterns often differ from guidelines, with certain therapies being underused or overused.^16,17,18^ Additionally, access to novel therapies can be limited by attrition, with a substantial number of patients with mCRPC historically receiving only 1 line of therapy.^19,20,21^ It is therefore important to examine clinical treatment patterns and attrition rates to determine whether treatment patterns have or have not shifted in this era of novel therapies. This information helps to inform clinical guidelines and future clinical trial designs and identifies needed areas of improvement in access and treatment sequencing. In this study, we aimed to characterize treatment patterns and attrition rates in a contemporary large dataset to understand how these therapies are used in clinical practice.

## Methods

### Study Design

This retrospective cohort study used the US-based electronic health record–derived, deidentified Flatiron Health Research Database,^22^ which includes patients treated at approximately 280 centers across the US, primarily in community oncology settings. Patient-level structured and unstructured data were deidentified and curated through technology-enabled abstraction in accordance with the Health Insurance Portability and Accountability Act. The University of Utah Institutional Review Board exempted the study from ethical approval and waived the need for informed consent due to the use of deidentified data. The study followed the Strengthening the Reporting of Observational Studies in Epidemiology (STROBE) reporting guideline.

### Patient Population

Patients with a diagnosis of mCRPC and valid first-line treatment initiated between January 1, 2021, and June 30, 2025, were eligible and were therefore included in the analysis. In this study, mCRPC diagnosis was confirmed by abstraction from patient documents according to Flatiron Health Research Database rules. Baseline characteristics, including age, race and ethnicity, sex, region, and insurance status, were collected from the electronic health record at the time of their first-line initiation. Race and ethnicity information was typically supplied through patient intake interviews and forms, although this could vary across practices. This information was then entered by clinical teams into deidentified electronic health records. Race and ethnicity categories included Asian, Hispanic or Latino, non-Hispanic Black, non-Hispanic White, other race or ethnicity (including American Indian or Alaska Native, Native Hawaiian or Other Pacific Islander, and multiracial), and unknown race or ethnicity; these data were included to allow comparison of self-reported race and ethnicity data to previously reported studies when interpreting results and generalizability of findings. Socioeconomic status was measured via an area level metric based on the Yost Index.^23^

### Outcomes

The study’s primary outcomes were the types of systemic therapies administered from first through fifth lines and the associated attrition rates among patients with mCRPC from 2021 to 2025. The definition of a line of therapy was the first eligible drug episode plus other eligible drugs given within 28 days of the start of the first agent. Treatment patterns were categorized into 1 of 9 groups: ARPIs, taxane-based therapies (with or without ARPIs), PARPI-based therapies (with or without ARPIs), platinum chemotherapy, Lu-177, radium Ra 223, sipuleucel-T, immunotherapy, and other (included agents that did not fit in any other category, including clinical trial drugs). Attrition was defined as the percentage of patients who did not receive the next line of therapy. This included patients who died, decided not to undergo further treatment, did not require another line of therapy in the study period, or were lost to follow-up from one line of therapy to the next. Secondary outcomes included treatment patterns by year of first-line initiation, disease state prior to mCRPC diagnosis, and treatments received prior to mCRPC diagnosis.

### Statistical Analysis

Therapies administered from the first- through fifth-line settings were summarized using frequencies and percentages. Subgroup analyses were conducted based on the year of therapy initiation for each line of treatment. All patient characteristics were summarized at the time of first-line initiation. Continuous variables are reported as medians and IQRs; categorical variables are reported as frequencies and percentages. To assess whether the proportion of patients receiving each first-line treatment category changed significantly across years, a χ^2^ test of independence was performed separately for each treatment category to compare the proportion of patients receiving that treatment vs all other treatments across years 2021 to 2025. A 2-sided *P* < .05 was considered statistically significant. All analyses were performed using R, version 4.5.1 (R Project for Statistical Computing).^24^

## Results

### Patient Demographics

**Figure 1.**
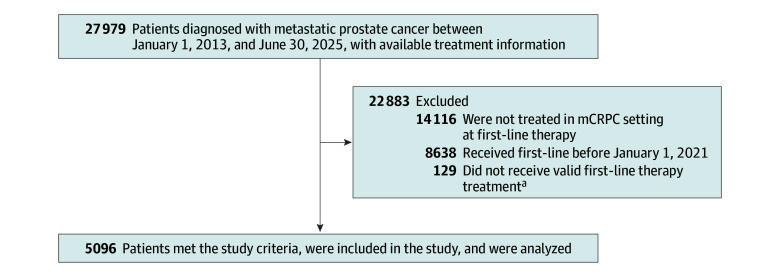
Patient Flow Diagram mCRPC indicates metastatic castration-resistant prostate cancer. ^a^Indicates treatment used in prostate cancer.

Of 27 979 patients with metastatic prostate cancer in the dataset, 5096 met the eligibility criteria and were included in the analysis (Figure 1). The median age was 75 (IQR, 68-82) years. Of these patients, 4039 (79.3%) experienced progression from mHSPC, 1019 (20.0%) experienced progression from nonmetastatic CRPC, and 38 (0.7%) experienced progression from nonmetastatic HSPC to mCRPC. A total of 86 patients (1.7%) were Asian, 380 (7.5%) were Hispanic or Latino, 616 (12.1%) were non-Hispanic Black, 2828 (55.5%) were non-Hispanic White, 370 (7.3%) were of other race or ethnicity, and 816 (16.0%) were of unknown race or ethnicity. Most patients (3888 [76.3%]) were treated in a community practice setting and had a commercial health plan (3130 [61.4%]). Overall patient characteristics, missing demographic information, and patient characteristics by the year of first-line therapy initiation are provided in eTable 1 in Supplement 1.

### Attrition Rates and Overall Treatment Patterns

Of the 5096 patients who received first-line therapy, 2731 (53.6%) received second-line therapy and 1334 (26.2%) received third-line therapy (eTable 2 in Supplement 1). Only 588 patients (11.5%) received fourth-line therapy and 226 (4.4%) received fifth-line therapy. ARPIs and taxanes were the most commonly used treatments. ARPI use decreased with each subsequent line. In the overall cohort, less than half of the patients (2192 [43.0%]) received a taxane-containing regimen. Use of Lu-177 and platinum-based therapies increased with each subsequent line.

### First-Line Treatment Patterns

**Figure 2.**
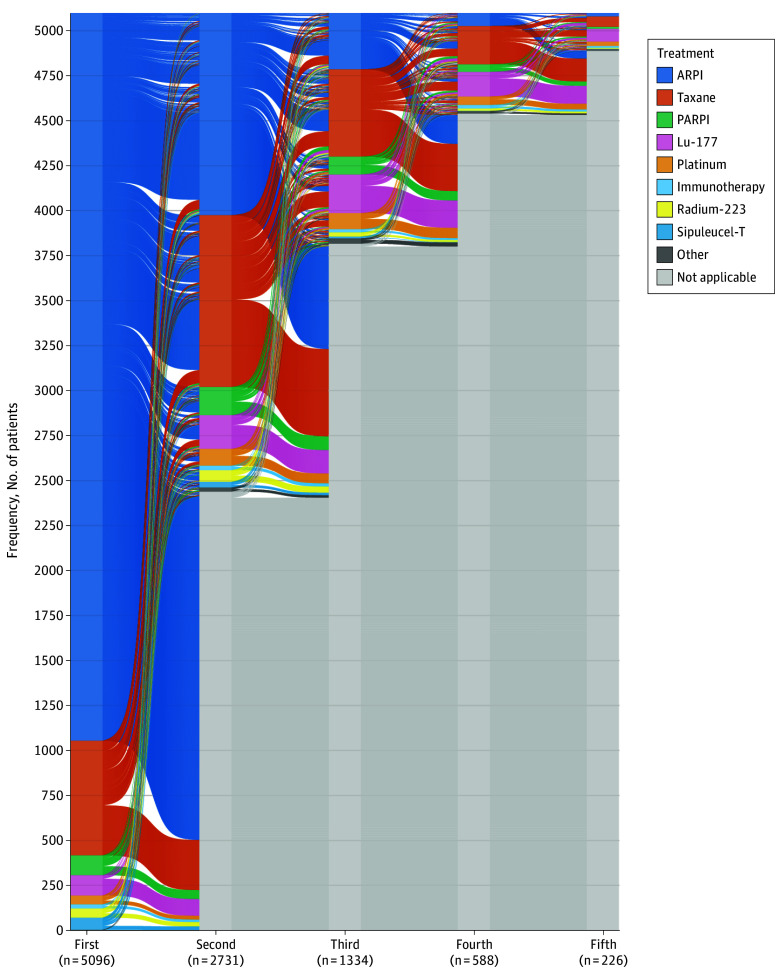
Sankey Diagram of Treatment Trends in First- Through Fifth-Line Therapy in Patients With Metastatic Castration-Resistant Prostate Cancer Taxane-based therapies includes taxane treatments with or without androgen receptor pathway inhibitors (ARPIs). Other includes agents that did not fit in any other category including clinical trial drugs. Lu-177 indicates lutetium Lu 177–prostate-specific membrane antigen 617–based therapies; PARPI, poly (adenosine diphosphate–ribose) polymerase inhibitor–based therapies with or without ARPI.

ARPIs were the most used first-line treatment (4041 patients [79.3%]), with taxane regimens being the second most common (637 patients [12.5%]). All other treatments were used infrequently in first-line treatment (<2.5%) (Figure 2 and eTable 2 in Supplement 1).

**Figure 3.**
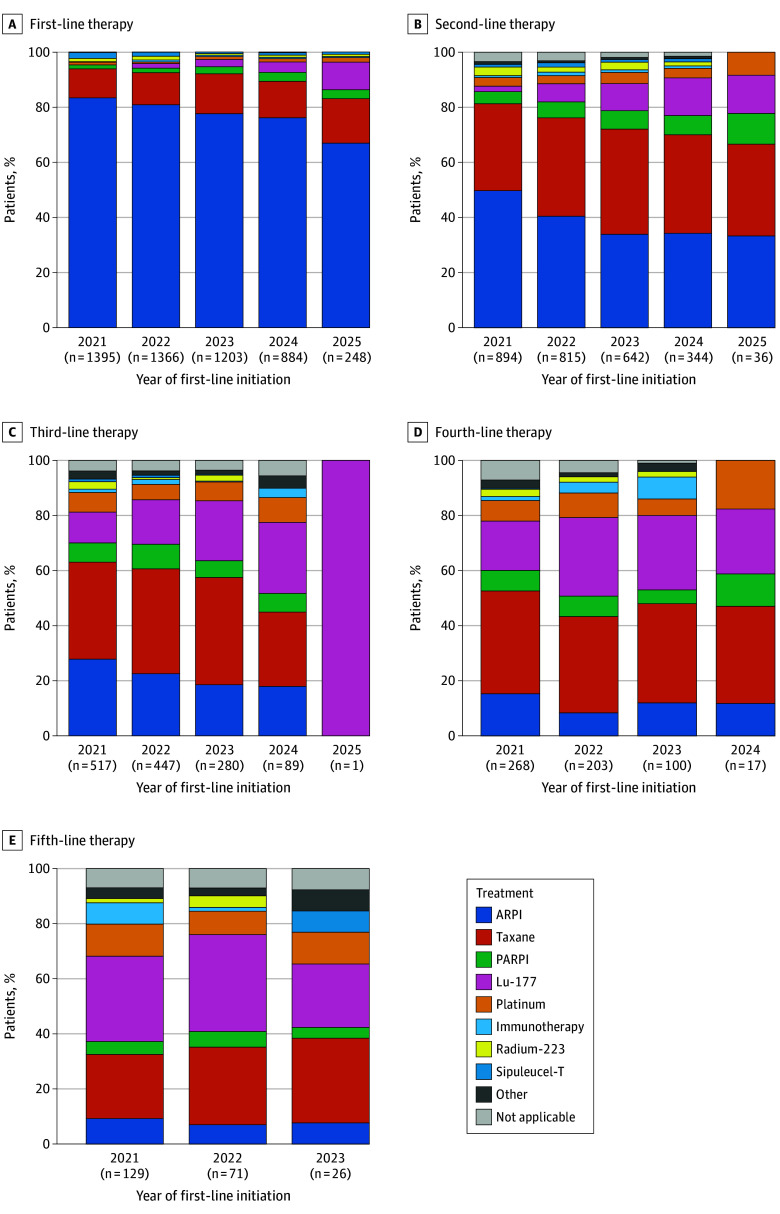
Bar Graphs Showing Treatment Patterns in First- Through Fifth-Line Treatment in Patients With Metastatic Castration-Resistant Prostate Cancer by Year of First-Line Treatment Initiation ARPIs indicates androgen receptor pathway inhibitors; Lu-177, lutetium Lu 177–prostate-specific membrane antigen 617–based therapies; and PARPI, poly (adenosine diphosphate–ribose) polymerase inhibitor–based therapies with or without ARPI.

ARPI use in first-line treatment varied significantly across the study period, decreasing from 1163 of 1395 patients (83.4%) in 2021 to 166 of 248 patients (66.9%) in 2025 (*P* < .001). The proportion of patients receiving Lu-177 as first-line treatment (113 of 5096 [2.2%]) differed significantly across years (*P* < .001), increasing from 0 of 1395 patients in 2021 to 25 of 248 patients (10.1%) in 2025 (Figure 3). First-line treatment patterns based on year of initiation from 2021 to 2025 are presented in eTable 3 in Supplement 1.

### Second-Line Treatment Patterns

ARPIs remained the most commonly used second-line treatment (1123 of 2731 patients [41.1%]), while use of taxanes (953 of 2731 [34.9%]), Lu-177 (187 of 2731 [6.8%]), and PARPIs (157 of 2731 patients [5.7%]) all numerically increased when moving from first- to second-line treatment (Figure 2 and eTable 2 in Supplement 1). From the 2021 cohort to the 2024 cohort, Lu-177 use in second-line settings numerically increased from 18 of 894 patients (2.0%) to 47 of 344 (13.7%). Proportional use of PARPIs in second-line treatment numerically increased from 39 of 894 patients (4.4%) in the 2021 cohort to 24 of 344 patients (7.0%) in the 2024 cohort. Concurrently, ARPI use in second-line treatment numerically decreased from 445 of 894 patients (49.8%) in the 2021 cohort to 118 of 344 (34.3%) in the 2024 cohort (Figure 3).

### Third-Line Treatment Patterns

Taxane-based regimens were used predominantly in third-line treatment (485 of 1334 [36.4%]), followed by ARPIs (313 of 1334 [23.5%]) and Lu-177 (215 of 1334 [16.1%]). PARPIs reached their highest proportional use in third-line treatment (99 of 1334 [7.4%]) (Figure 2 and eTable 2 in Supplement 1).

Use of Lu-177 in third-line treatment numerically increased over time, rising from 58 of 517 patients (11.2%) in the 2021 cohort to 23 of 89 patients (25.8%) in the 2024 cohort, while PARPI use in third-line treatment was numerically similar for the 2021 (36 of 517 [7.0%]) and the 2024 (6 of 89 [6.7%]) cohorts. Taxane-based regimens in third-line treatment numerically decreased from 182 of 517 patients (35.2%) in the 2021 cohort to 24 of 89 patients (27.0%) in the 2024 cohort (Figure 2).

### Fourth- and Fifth-Line Treatment Patterns

Taxane regimens remained the most common treatment in fourth-line treatment (213 of 588 [36.2%]). Lu-177 became the second most used fourth-line treatment (137 of 588 [23.3%]), with ARPIs (72 of 588 [12.2%]) and platinum chemotherapy (47 of 588 [8.0%]) being the next most common treatments. In fifth-line treatment, Lu-177 was used predominantly (71 of 226 patients [31.4%]), with taxane regimens being the next most common treatment (58 of 226 [25.7%]) (Figure 2 and eTable 2 in Supplement 1).

### Treatment Patterns Based on Disease State Prior to mCRPC Diagnosis

Among patients with progression to mCRPC from mHSPC (4039 patients), 3206 (79.4%) received an ARPI and 503 (12.5%) received a taxane as first-line treatment in the mCRPC setting. Among those with progression from nonmetastatic CRPC (1019 patients), 809 (79.4%) received an ARPI and 126 (12.4%) received a taxane in first-line mCRPC treatment (eFigure in Supplement 1).

### Treatment Patterns Based on Treatments Prior to mCRPC Setting

Of the 5096 total patients, 1099 (21.6%) had evidence of receiving systemic treatment beyond androgen deprivation therapy prior to mCRPC diagnosis. Among the 705 patients (64.1%) who previously were treated with a regimen containing an ARPI, 500 (70.9%) received an ARPI-containing regimen as first-line mCRPC treatment, and 110 (15.6%) received a taxane-only regimen. Among the 332 patients (30.2%) who had received taxane therapy before mCRPC, 282 (84.9%) were treated with an ARPI-containing regimen as first-line treatment and 21 (6.3%) received a taxane regimen.

## Discussion

In this analysis, nearly half of patients did not receive second-line therapy, with attrition approaching 75% by the third-line setting. ARPIs remained the most frequently used treatments in first- and second-line settings, while taxanes were the most commonly used therapy in third- and fourth-line settings. Less than half of the patients received a taxane-containing regimen. Use of PARPIs and Lu-177 increased in the later lines of therapy.

The attrition rates seen in our study are consistent with those in prior research.^16,21,25^ Attrition in mCRPC occurs for multiple reasons, including patient preference, toxicity, disease progression, financial burden, patient frailty, or death from cancer or other causes.^26,27^ One study of patients with Medicare found death was a common cause of attrition, with 52% to 62% of patients dying between each line of therapy.^16^ Even if a patient does not die, impact from mCRPC and the treatment itself can contribute to attrition. For example, a retrospective study on Lu-177 found that disease progression and serious adverse events were among the most common reasons for treatment discontinuation.^28^ Progressive disease symptoms along with cumulative toxicity from prior treatments often make it challenging for patients to continue to another line of therapy. Further research is needed to better understand the contributors to attrition in mCRPC, and improved strategies to improve access are critical, as delivering the most effective first-line treatment is essential to ensure patients benefit before they are lost to attrition.

ARPI use as the predominant first-line mCRPC therapy in our study aligns with previous findings.^16,17,18,25^ We also noted a decline in first-line ARPI use from 2021 to 2025, likely reflecting increasing use of other treatments. However, 70.9% of patients previously treated with an ARPI received another ARPI as first-line mCRPC treatment, indicating that ARPI switch remains common in clinical practice, despite evidence suggesting reduced effectiveness with this approach.^29,30^ In unselected populations, chemotherapy or Lu-177 are currently available options for patients whose disease progresses during treatment with an ARPI. Few previous studies have reported use of Lu-177 in mCPRC.^18^ In this analysis, taxanes are the second most common therapy after ARPIs in first- and second-line mCRPC therapy, indicating reliance on chemotherapy following progression during ARPI therapy in a clinical setting. However, in the third- to fifth-line settings, Lu-177 use increased from 2021 to 2024, and our data also showed a statistically significant variation in Lu-177 use as first-line treatment, with use increasing from 2021 to 2025. It is possible that first-line use of Lu-177 in mCRPC will continue to increase following recent trial findings^15^ and the US Food and Drug Administration approval in 2025 for use of Lu-177 in patients who have experienced disease progression during ARPI treatment.^31^ Results from the phase 2 trial Canadian Cancer Trials Group Study PR.21 comparing docetaxel with Lu-177 in chemotherapy-naive patients with disease progression during prior ARPI therapy showed that Lu-177 did not improve radiographic progression-free survival, despite leading to higher prostate-specific antigen response rates and less grade 3 toxicity or greater.^32^ Moreover, crossover from docetaxel to Lu-177 was more common than the reverse. This highlights the importance of treatment sequencing, although the optimal sequencing of chemotherapy and Lu-177 remains to be explored. Use of PARPI was limited throughout all lines of therapy and peaked in the third-line setting, when approximately 75% of patients had already been lost to attrition. It is possible that recent approvals of PARPI and ARPI combinations will lead to earlier use of PARPI in mCRPC.^12,13,14^ One of the possible reasons for the lack of early use is due to the low rate of next-generation DNA sequencing testing. Despite the recommendation for universal germline and somatic tumor testing for patients with metastatic prostate cancer,^8,33,34,35^ previous studies showed that most patients with mCRPC do not receive testing for *HRR* gene mutations.^36,37,38^ Additionally, when genetic testing is completed, it is often completed weeks after first-line therapy has already been started, although this has been improving in recent years.^36^ The high attrition rate in this study reinforces the need for genetic testing to be completed before the diagnosis of mCRPC to allow for the best sequencing of lines of therapy, including earlier use of PARPIs in patients with *HRR* gene mutations.^1,39,40,41^

Treatment patterns in the mCRPC setting will likely continue to evolve and become more complex. One reason is due to the expanding mHSPC treatment landscape. Agents such as PARPIs and Lu-177 will likely be used in the mHSPC setting for select patient populations based on the results of recent trial findings^42,43^ altering later first-line mCRPC treatment decisions. Additionally, new potential additional therapeutic agents such as capivasertib in mHSPC may further alter later treatment sequencing in mCRPC.^44^

### Strengths and Limitations

The strengths of this study include its use of patient-level data from a large multicenter nationwide database that reflects contemporary clinical practice. These data can directly inform control arm selection and trial design for upcoming studies in the mCRPC setting. There are also several limitations to this study, including its retrospective nature that can be impacted by selection bias. Additionally, there was incomplete or no characterization of some data regarding baseline and clinical characteristics (disease burden, location of metastatic sites), patient and physicians’ preferences, and reasons for attrition. These unknown factors are potential confounding factors that were not controlled for in this study. Patients in our study were US based, were mainly treated in community practices, and mostly had commercial health insurance, which may impact the generalizability of the findings. This study was descriptive of treatment patterns and did not report treatment outcomes or adverse event data, which may also impact treatment sequencing.

## Conclusions

In this large cohort study of patients with mCRPC, only approximately half received second-line therapy, with a steep drop-off across subsequent lines. ARPIs and taxanes dominated first-line treatment, whereas Lu-177 and PARPI-based regimens were most commonly used in later lines of treatment. Despite major therapeutic advances and the availability of multiple effective drug classes, clinical treatment patterns in mCRPC revealed substantial underuse and high attrition. These findings underscore the growing complexity of mCRPC care, highlight critical gaps in access and sequencing that may limit the clinical impact of therapeutic innovation, and have direct implications for health policy and the design of future clinical trials.

## Notes

Supplement 1. **eTable 1**. Baseline Patient Demographic Characteristics Overall and by Year of First-Line Initiation in Patients With mCRPC **eTable 2.** Treatment Patterns and Attrition Rates Across 5 Lines of Therapy for Patients With mCRPC **eTable 3.** Treatment Patterns in mCRPC in First Line Based on Year of Initiation From 2021 to 2025 **eFigure.** Treatment Patterns in First Line Based on Disease Setting Before mCRPC Diagnosis

Supplement 2.
Data Sharing Statement

## References

[1] Saeed F, Berchuck JE, Bilen MA, . Optimizing treatment for metastatic castration-resistant prostate cancer: Food and Drug Administration-approved therapies, emerging strategies, and biomarker-driven approaches. Cancer. 2025;131(16):e70037. doi:10.1002/cncr.70037 40782341

[2] Tannock IF, de Wit R, Berry WR, ; TAX 327 Investigators. Docetaxel plus prednisone or mitoxantrone plus prednisone for advanced prostate cancer. N Engl J Med. 2004;351(15):1502-1512. doi:10.1056/NEJMoa040720 15470213

[3] de Bono JS, Oudard S, Ozguroglu M, ; TROPIC Investigators. Prednisone plus cabazitaxel or mitoxantrone for metastatic castration-resistant prostate cancer progressing after docetaxel treatment: a randomised open-label trial. Lancet. 2010;376(9747):1147-1154. doi:10.1016/S0140-6736(10)61389-X 20888992

[4] Kantoff PW, Higano CS, Shore ND, ; IMPACT Study Investigators. Sipuleucel-T immunotherapy for castration-resistant prostate cancer. N Engl J Med. 2010;363(5):411-422. doi:10.1056/NEJMoa1001294 20818862

[5] de Bono JS, Logothetis CJ, Molina A, ; COU-AA-301 Investigators. Abiraterone and increased survival in metastatic prostate cancer. N Engl J Med. 2011;364(21):1995-2005. doi:10.1056/NEJMoa1014618 21612468 PMC3471149

[6] Scher HI, Fizazi K, Saad F, ; AFFIRM Investigators. Increased survival with enzalutamide in prostate cancer after chemotherapy. N Engl J Med. 2012;367(13):1187-1197. doi:10.1056/NEJMoa1207506 22894553

[7] Parker C, Nilsson S, Heinrich D, ; ALSYMPCA Investigators. Alpha emitter radium-223 and survival in metastatic prostate cancer. N Engl J Med. 2013;369(3):213-223. doi:10.1056/NEJMoa1213755 23863050

[8] Garje R, Riaz IB, Naqvi SAA, . Systemic therapy in patients with metastatic castration-resistant prostate cancer: ASCO guideline update. J Clin Oncol. 2025;43(20):2311-2334. doi:10.1200/JCO-25-00007 40315400

[9] de Bono J, Mateo J, Fizazi K, . Olaparib for metastatic castration-resistant prostate cancer. N Engl J Med. 2020;382(22):2091-2102. doi:10.1056/NEJMoa1911440 32343890

[10] Fizazi K, Piulats JM, Reaume MN, ; TRITON3 Investigators. Rucaparib or physician’s choice in metastatic prostate cancer. N Engl J Med. 2023;388(8):719-732. doi:10.1056/NEJMoa2214676 36795891 PMC10064172

[11] Sartor O, de Bono J, Chi KN, ; VISION Investigators. Lutetium-177-PSMA-617 for metastatic castration-resistant prostate cancer. N Engl J Med. 2021;385(12):1091-1103. doi:10.1056/NEJMoa2107322 34161051 PMC8446332

[12] Agarwal N, Azad AA, Carles J, . Talazoparib plus enzalutamide in men with first-line metastatic castration-resistant prostate cancer (TALAPRO-2): a randomised, placebo-controlled, phase 3 trial. Lancet. 2023;402(10398):291-303. doi:10.1016/S0140-6736(23)01055-3 37285865

[13] Chi KN, Rathkopf D, Smith MR, ; MAGNITUDE Principal Investigators. Niraparib and abiraterone acetate for metastatic castration-resistant prostate cancer. J Clin Oncol. 2023;41(18):3339-3351. doi:10.1200/JCO.22.01649 36952634 PMC10431499

[14] Clarke NW, Armstrong AJ, Thiery-Vuillemin A, . Abiraterone and olaparib for metastatic castration-resistant prostate cancer. NEJM Evid. 2022;1(9):a2200043. doi:10.1056/EVIDoa2200043 38319800

[15] Morris MJ, Castellano D, Herrmann K, ; PSMAfore Investigators. ^177^Lu-PSMA-617 versus a change of androgen receptor pathway inhibitor therapy for taxane-naive patients with progressive metastatic castration-resistant prostate cancer (PSMAfore): a phase 3, randomised, controlled trial. Lancet. 2024;404(10459):1227-1239. doi:10.1016/S0140-6736(24)01653-2 39293462 PMC12121614

[16] Freedland SJ, Davis M, Epstein AJ, Arondekar B, Ivanova JI. Real-world treatment patterns and overall survival among men with metastatic castration-resistant prostate cancer (mCRPC) in the US Medicare population. Prostate Cancer Prostatic Dis. 2024;27(2):327-333. doi:10.1038/s41391-023-00725-8 37783836 PMC11096091

[17] Barata PC, Leith A, Ribbands A, . Real-world treatment patterns among patients with metastatic castration-resistant prostate cancer: results from an international study. Oncologist. 2023;28(9):e737-e747. doi:10.1093/oncolo/oyad046 37014097 PMC10485288

[18] Raval AD, Queen V, Korn MJ, Quintero V, Freedland SJ. Real-world treatment patterns and survival in metastatic castration-resistant prostate cancer: a systematic review of observational studies. Eur Urol Focus. 2026;12(2):284-296. doi:10.1016/j.euf.2025.10.011 41266184

[19] Moldaver DM, Hassan S, Seung SJ, Edwin J, Clouthier DL, Vera-Badillo FE. A real-world retrospective analysis of the management of metastatic castrate-resistant prostate cancer in Ontario, Canada from 2010 - 2018. Urol Oncol. 2023;41(3):146.e13-146.e22. doi:10.1016/j.urolonc.2022.11.019 36641303

[20] Shayegan B, Wallis CJD, Malone S, . Real-world use of systemic therapies in men with metastatic castration resistant prostate cancer (mCRPC) in Canada. Urol Oncol. 2022;40(5):192.e1-192.e9. doi:10.1016/j.urolonc.2022.01.009 35216890

[21] Shore ND, Laliberté F, Ionescu-Ittu R, . Real-world treatment patterns and overall survival of patients with metastatic castration-resistant prostate cancer in the US prior to PARP inhibitors. Adv Ther. 2021;38(8):4520-4540. doi:10.1007/s12325-021-01823-6 34282527 PMC8342357

[22] Database characterization guide. Flatiron Health. Published March 18, 2025. Accessed October 10, 2025. https://flatiron.com/database-characterization

[23] Yost K, Perkins C, Cohen R, Morris C, Wright W. Socioeconomic status and breast cancer incidence in California for different race/ethnic groups. Cancer Causes Control. 2001;12(8):703-711. doi:10.1023/A:1011240019516 11562110

[24] *R: A Language and Environment for Statistical Computing*. Version 4.5.1. R Foundation for Statistical Computing. Accessed October 16, 2025. https://www.R-project.org/

[25] Papet J, Augusto-Pelegrin L, Christy F, . Real-world treatment patterns and survival outcomes in patients with metastatic castration-resistant prostate cancer in France: lessons from the prospective OPALE study. Anticancer Drugs. 2025;36(10):805-811. doi:10.1097/CAD.0000000000001756 41082589

[26] Chakrani Z, Mellgard G, Saffran N, . Risk factors for early treatment discontinuation due to toxicity among patients with metastatic castration-resistant prostate cancer receiving androgen receptor-targeted therapy. Am J Clin Oncol. 2024;47(6):271-278. doi:10.1097/COC.0000000000001087 38344754

[27] Swami U, Aggarwal H, Zhou M, . Treatment patterns, clinical outcomes, health care resource utilization and costs in older patients with metastatic castration-resistant prostate cancer in the United States: an analysis of SEER-Medicare data. Clin Genitourin Cancer. 2023;21(5):517-529. doi:10.1016/j.clgc.2023.04.014 37248148

[28] Samadi MH, Shamshirgaran A, Sahafi P, . Treatment discontinuation in metastatic castration-resistant prostate cancer (mCRPC) patients treated with lutetium-177 (^177^Lu)-PSMA-617: a retrospective real-world study. Clin Genitourin Cancer. 2026;24(2):102499. doi:10.1016/j.clgc.2025.102499 41620317

[29] de Wit R, de Bono J, Sternberg CN, ; CARD Investigators. Cabazitaxel versus abiraterone or enzalutamide in metastatic prostate cancer. N Engl J Med. 2019;381(26):2506-2518. doi:10.1056/NEJMoa1911206 31566937

[30] Narayan V, Patel MY, Teitsson S, . Treatment patterns and survival outcomes among androgen receptor pathway inhibitor-experienced patients with metastatic castration-resistant prostate cancer. Clin Genitourin Cancer. 2024;22(6):102188. doi:10.1016/j.clgc.2024.102188 39232487

[31] FDA expands Pluvicto’s metastatic castration-resistant prostate cancer indication. US Food and Drug Administration. Accessed April 12, 2026. https://www.fda.gov/drugs/resources-information-approved-drugs/fda-expands-pluvictos-metastatic-castration-resistant-prostate-cancer-indication

[32] Chi KN, Saad F, Ding K, . LBA89: a randomized phase II study of 177Lu-PSMA-617 vs docetaxel in patients with metastatic castration-resistant prostate cancer (mCRPC) and PSMA-positive disease: Canadian Cancer Trials Group (CCTG) study PR.21. Ann Oncol. 2025;36(suppl 2):S1745-S1746. doi:10.1016/j.annonc.2025.09.105

[33] NCCN clinical practice guidelines in oncology prostate cancer. National Comprehensive Cancer Network. Accessed November 7, 2025. https://www.nccn.org/guidelines/guidelines-detail?category=1&id=1459 10.6004/jnccn.2010.0012 20141676

[34] Armstrong AJ, Taylor A, Haffner MC, . Germline and somatic testing for homologous repair deficiency in patients with prostate cancer (part 1 of 2). Prostate Cancer Prostatic Dis. 2025;28(3):652-661. doi:10.1038/s41391-024-00901-4 39354185 PMC12399426

[35] Yu EY, Rumble RB, Agarwal N, . Germline and somatic genomic testing for metastatic prostate cancer: ASCO guideline. J Clin Oncol. 2025;43(6):748-758. doi:10.1200/JCO-24-02608 39787437

[36] Barata PC, Assayag J, Li B, Siu G, Niyazov A. Genetic testing in men with metastatic castration-resistant prostate cancer. JAMA Oncol. 2024;10(7):975-977. doi:10.1001/jamaoncol.2024.0851 38696212 PMC11066759

[37] Castro E, Orji C, Ribbands A, . Real-world treatment patterns and genetic testing in a metastatic castration-resistant prostate cancer setting in Europe. Future Oncol. 2025;21(9):1085-1099. doi:10.1080/14796694.2025.2470616 40105456 PMC11988269

[38] Hage Chehade C, Jo Y, Gebrael G, . Trends and disparities in next-generation sequencing in metastatic prostate and urothelial cancers. JAMA Netw Open. 2024;7(7):e2423186. doi:10.1001/jamanetworkopen.2024.23186 39023888 PMC11258596

[39] Jiang B, Wang B, Chen Y, Chen Y, Li B, Bi J. Comparative therapeutic efficacy and safety of first-line and second-line therapies for metastatic castration-resistant prostate cancer: a systematic review and network meta-analysis. EClinicalMedicine. 2025;81:103129. doi:10.1016/j.eclinm.2025.103129 40104085 PMC11914769

[40] Francini E, Agarwal N, Castro E, . Intensification approaches and treatment sequencing in metastatic castration-resistant prostate cancer: a systematic review. Eur Urol. 2025;87(1):29-46. doi:10.1016/j.eururo.2024.09.008 39306478 PMC13007795

[41] Ostrowski M, Jo Y, Hage Chehade C, . Receipt of PARP inhibitors in patients with metastatic prostate cancer harboring BRCA1/2 alterations. JAMA Netw Open. 2025;8(10):e2534968. doi:10.1001/jamanetworkopen.2025.34968 41037269 PMC12492053

[42] Attard G, Agarwal N, Graff JN, . Niraparib and abiraterone acetate plus prednisone for HRR-deficient metastatic castration-sensitive prostate cancer: a randomized phase 3 trial. Nat Med. 2025;31(12):4109-4118. doi:10.1038/s41591-025-03961-8 41057655 PMC12705445

[43] Tagawa ST, Sartor O, Piulats JM, . LBA6 phase III trial of [177Lu]Lu-PSMA-617 combined with ADT + ARPI in patients with PSMA-positive metastatic hormone-sensitive prostate cancer (PSMAddition). Ann Oncol. 2025;36:S1627-S1628. doi:10.1016/j.annonc.2025.09.101

[44] Fizazi K, Clarke NW, De Santis M, ; CAPItello-281 Study Group. Capivasertib plus abiraterone in PTEN-deficient metastatic hormone-sensitive prostate cancer: CAPItello-281 phase III study. Ann Oncol. 2026;37(1):53-68. doi:10.1016/j.annonc.2025.10.004 41120017

