# Supplementary material for: Treatment Patterns and Attrition in Metastatic Castration-Resistant Prostate Cancer
Source: JAMA Netw Open. 2026 Jun 29;9(6):e2620750. doi: 10.1001/jamanetworkopen.2026.20750 (PMC13316606; doi:10.1001/jamanetworkopen.2026.20750)
Supplement: Supplement 1. — eTable 1. Baseline Patient Demographic Characteristics Overall and by Year of First-Line Initiation in Patients With mCRPC eTable 2. Treatment Patterns and Attrition Rates Across 5 Lines of Therapy for Patients With mCRPC eTable 3. Treatment Patterns in mCRPC in First Line Based on Year of Initiation From 2021 to 2025 eFigure. Treatment Patterns in First Line Based on Disease Setting Before mCRPC Diagnosis [file jamanetwopen-e2620750-s001.pdf]

Supplementary Online Content

Hooper G, Jo Y, Nandakumar V, et al. Treatment patterns and attrition in metastatic castration-resistant prostate cancer. *JAMA Netw Open.* 2026;9(6):e2620750. doi:10.1001/jamanetworkopen.2026.20750

eTable 1. Baseline Patient Demographic Characteristics Overall and by Year of First-Line Initiation in Patients With mCRPC

eTable 2. Treatment Patterns and Attrition Rates Across 5 Lines of Therapy for Patients With mCRPC

eTable 3. Treatment Patterns in mCRPC in First Line Based on Year of Initiation From 2021 to 2025

eFigure. Treatment Patterns in First Line Based on Disease Setting Before mCRPC Diagnosis

This supplementary material has been provided by the authors to give readers additional information about their work.

© 2026 Hooper G et al. *JAMA Network Open*.

eTable 1. Baseline Patient Demographic Characteristics Overall and by Year of First-Line Initiation in Patients With mCRPC

**Characteristic**	**Overall** N = 5,096[1]	**2021** N = 1,395[1]	**2022** N = 1,366[1]	**2023** N = 1,203[1]	**2024** N = 884[1]	**2025** N = 248[1]
**Age**	75 (68,82)	75 (67,81)	74 (67,81)	75 (68,82)	76 (70,83)	76 (69,84)
**Race/Ethnicity**						
Asian, Non-Hispanic	86 (1.7)	20 (1.4)	23 (1.7)	23 (1.9)	14 (1.6)	6 (2.4)
Black, Non-Hispanic	616 (12.1)	160 (11.5)	169 (12.4)	157 (13.1)	104 (11.8)	26 (10.5)
Hispanic Latino	380 (7.5)	109 (7.8)	93 (6.8)	95 (7.9)	66 (7.5)	17 (6.9)
White, Non-Hispanic	2,828 (55.5)	748 (53.6)	790 (57.8)	647 (53.8)	504 (57.0)	139 (56.1)
Other[2]	370 (7.3)	145 (10.4)	87 (6.4)	74 (6.2)	50 (5.7)	14 (5.7)
Unknown	816 (16.0)	213 (15.3)	204 (14.9)	207 (17.2)	146 (16.5)	46 (18.6)
**Sex**						
Male	5,096 (100.0)	1,395 (100.0)	1,366 (100.0)	1,203 (100.0)	884 (100.0)	248 (100.0)
Female	0 (0.0)	0 (0.0)	0 (0.0)	0 (0.0)	0 (0.0)	0 (0.0)
**Region**						
Northeast	626 (12.3)	177 (12.7)	176 (12.9)	136 (11.3)	103 (11.7)	34 (13.7)
Midwest	461 (9.1)	130 (9.3)	122 (8.9)	103 (8.6)	88 (10.0)	18 (7.3)
South	2,002 (39.3)	535 (38.4)	522 (38.2)	494 (41.1)	352 (39.8)	99 (39.9)
West	803 (15.8)	227 (16.3)	210 (15.4)	184 (15.3)	144 (16.3)	38 (15.3)
Unknown or Unclassified	1,204 (23.6)	326 (23.4)	336 (24.6)	286 (23.8)	197 (22.3)	59 (23.8)

© 2026 Hooper G et al. *JAMA Network Open*.

**SES[a]**						
1 - Lowest SES	768 (15.1)	187 (13.4)	205 (15.0)	195 (16.2)	144 (16.3)	37 (14.9)
2	873 (17.1)	247 (17.7)	229 (16.8)	210 (17.5)	160 (18.1)	27 (10.9)
3	955 (18.7)	271 (19.4)	249 (18.2)	220 (18.3)	157 (17.8)	58 (23.4)
4	1,120 (22.0)	312 (22.4)	298 (21.8)	250 (20.8)	202 (22.9)	58 (23.4)
5 - Highest SES	944 (18.5)	241 (17.3)	269 (19.7)	223 (18.5)	158 (17.9)	53 (21.4)
Unknown	436 (8.6)	137 (9.8)	116 (8.5)	105 (8.7)	63 (7.1)	15 (6.1)
**Practice Type**						
Academic	803 (15.8)	206 (14.8)	232 (17.0)	188 (15.6)	138 (15.6)	39 (15.7)
Community	3,888 (76.3)	1,078 (77.3)	996 (72.9)	926 (77.0)	694 (78.5)	194 (78.2)
Unknown	405 (8.0)	111 (8.0)	138 (10.1)	89 (7.4)	52 (5.9)	15 (6.1)
**Insurance**						
Commercial Health Plan	3,130 (61.4)	816 (58.5)	835 (61.1)	733 (60.9)	571 (64.6)	175 (70.6)
Medicare/Other Government Program	921 (18.1)	220 (15.8)	261 (19.1)	233 (19.4)	163 (18.4)	44 (17.8)
Others	431 (8.5)	135 (9.7)	110 (8.1)	94 (7.8)	72 (8.1)	20 (8.1)
Unknown	614 (12.1)	224 (16.1)	160 (11.7)	143 (11.9)	78 (8.8)	9 (3.6)

[1] Median (Q1, Q3); n (%) [2] Other races include Alaska Native, American Indian, Native Hawaiian, Other Pacific Islander who are not Hispanic or Latino, or multiracial. [a] Socioeconomic status.

© 2026 Hooper G et al. *JAMA Network Open*.

eTable 2. Treatment Patterns and Attrition Rates Across 5 Lines of Therapy for Patients With mCRPC

**Treatments, No. (%)**	**First line N = 5,096 (100)**	**Second line N = 2,731 (53.6)**	**Third line N = 1,334 (26.2)**	**Fourth line N = 588 (11.5)**	**Fifth line N = 226 (4.4)**
ARPIs	4041 (79.3)	1123 (41.1)	313 (23.5)	72 (12.2)	19 (8.4)
Taxane[a]	637 (12.5)	953 (34.9)	485 (36.4)	213 (36.2)	58 (25.7)
Lu-177	113 (2.2)	187 (6.9)	215 (16.1)	137 (23.3)	71 (31.4)
PARPi[b]	110 (2.2)	157 (5.8)	99 (7.4)	42 (7.1)	11 (4.9)
Sipuleucel-T	67 (1.3)	32 (1.2)	10 (0.8)	1 (0.2)	2 (0.9)
Radium-223	51 (1.0)	66 (2.4)	23 (1.7)	13 (2.2)	5 (2.2)
Platinum	49 (1.0)	93 (3.4)	89 (6.7)	47 (8.0)	24 (10.6)
Immunotherapy	23 (0.5)	25 (0.9)	18 (1.4)	20 (3.4)	11 (4.9)
Other[c]	5 (0.1)	95 (3.5)	82 (6.2)	43 (7.3)	25 (11.1)

Abbreviations: ARPIs, androgen receptor pathway inhibitors; PARPi, Poly (ADP-ribose) polymerase inhibitors-based therapies with or without ARPI; Lu-177, ^{177}Lu-PSMA-617-based therapies. [a] Included taxane treatments with or without ARPI. [b] Included PARPi treatments with or without ARPI. [c] Other included agents that did not fit in any other category including clinical trial drugs.

© 2026 Hooper G et al. *JAMA Network Open*.

eTable 3. Treatment Patterns in mCRPC in First Line Based on Year of Initiation From 2021 to 2025

Treatment	**2021** N = 1,395[1]	**2022** N = 1,366[1]	**2023** N = 1,203[1]	**2024** N = 884[1]	**2025** N = 248[1]	**p-value**[2]
ARPI	1163 (83.4)	1105 (80.9)	934 (77.6)	673 (76.1)	166 (66.9)	<0.001
Taxane	147 (10.5)	159 (11.6)	174 (14.5)	117 (13.2)	40 (16.1)	0.009
Lu-177	0 (0.0)	23 (1.7)	32 (2.7)	33 (3.7)	25 (10.1)	<0.001
PARPi	20 (1.4)	22 (1.6)	31 (2.6)	29 (3.3)	8 (3.2)	0.011
Sipuleucel-T	30 (2.2)	20 (1.5)	7 (0.6)	8 (0.9)	2 (0.8)	0.007
Radium-223	17 (1.2)	18 (1.3)	7 (0.6)	7 (0.8)	2 (0.8)	0.3
Platinum	14 (1.0)	8 (0.6)	12 (1.0)	11 (1.2)	4 (1.6)	0.3
Immunotherapy	2 (0.1)	10 (0.7)	6 (0.5)	4 (0.5)	1 (0.4)	0.2
Other	2 (0.1)	1 (0.1)	0 (0.0)	2 (0.2)	0 (0.0)	0.5

[1] n (%). [2] Pearson's Chi-squared test

© 2026 Hooper G et al. *JAMA Network Open*.

eFigure. Treatment Patterns in First Line Based on Disease Setting Before mCRPC Diagnosis

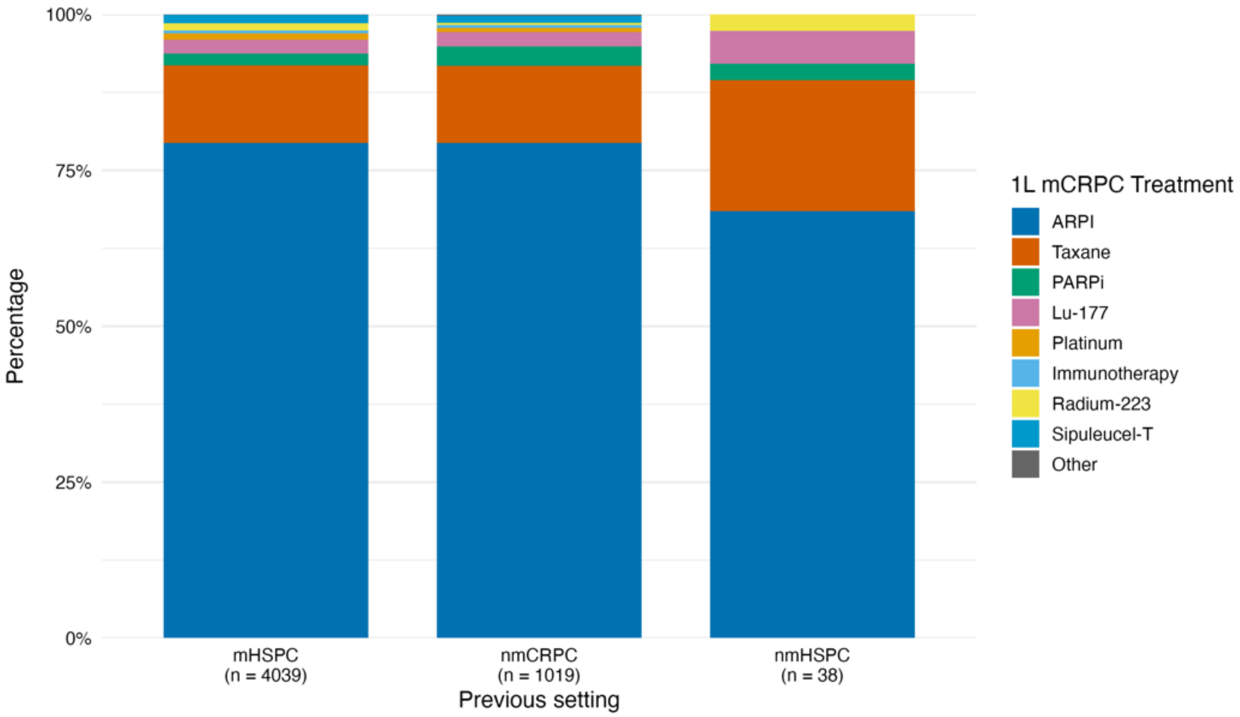

Abbreviations: 1L, first-line treatment; mHSPC, metastatic hormone-sensitive prostate cancer; nmCRPC, non-metastatic castration-resistant prostate cancer; nmHSPC, non-metastatic hormone-sensitive prostate cancer; ARPI, androgen receptor pathway inhibitors; PARPi, Poly (ADP-ribose) polymerase inhibitors-based therapies with or without ARPI; Lu-177, ^{177}Lu-PSMA-617-based therapies. Other included agents that did not fit in any other category including clinical trial drugs.

© 2026 Hooper G et al. *JAMA Network Open*.
