# Supplementary material for: Treatment Patterns and Attrition in Metastatic Castration-Resistant Prostate Cancer
Source: JAMA Netw Open. 2026 Jun 29;9(6):e2620750. doi: 10.1001/jamanetworkopen.2026.20750 (PMC13316606; doi:10.1001/jamanetworkopen.2026.20750)
Supplement: Supplement 2. — Data Sharing Statement [file jamanetwopen-e2620750-s002.pdf]

Data Sharing Statement

Hooper. Treatment Patterns and Attrition in Metastatic Castration-Resistant Prostate Cancer. *JAMA Netw Open*. Published June 29, 2026. doi:10.1001/jamanetworkopen.2026.20750

Data
Data available: No

Additional Information
Explanation for why data not available: The data that support the findings of this study were originated by and are the property of Flatiron Health, Inc. Requests for data sharing by license or by permission for the specific purpose of replicating results in this manuscript can be submitted to PublicationsDataAccess@flatiron.com.
